# Clinical Efficiency of Two Sequences of Orthodontic Wires to Correct Crowding of the Lower Anterior Teeth

**DOI:** 10.1155/2015/690280

**Published:** 2015-06-14

**Authors:** Cláudia Maria de Castro Serafim, Júlio de Araújo Gurgel, Carollyne Mota Tiago, Rudys Rodolfo de Jesus Tavarez, Etevaldo Matos Maia Filho

**Affiliations:** ^1^Ceuma University, Rua Josué Montello No. 1, Renascença II, 65075-120 São Luís, MA, Brazil; ^2^FACIT Dental School, Rua D 25, Qd 11, Lt 10, Avenida José de Brito George Yunes, 77818-650 Araguaína, TO, Brazil

## Abstract

This study compared time to correction of mandibular anterior crowding using two arch wire sequences, one with conventional nickel-titanium (NiTi) arch wires and the other with conventional and NiTi heat-activated arch wires. Twenty-two boys and girls (mean age: 16.68 ± 2.66) with moderate crowding (3–6 mm) were assigned randomly to one of two groups and followed up for five months (six assessments) when arch wires were changed. Time to crowding correction was analyzed statistically using the Kaplan-Meier method. Data were collected during the five-month follow-up, and time to correction was compared between groups using the log rank test. At the end of follow-up, mandibular crowding was corrected in 100% of the cases in the group treated with the sequence that included NiTi heat-activated arch wires, whereas about 30% of those treated with NiTi arch wires were not completely corrected. There was a significant difference in time to complete treatment between groups (log rank = 5.996; *p* < 0.05). In the group treated with the sequence that included heat-activated wires, alignment and leveling of mandibular anterior teeth were completed earlier than in the group treated only with conventional NiTi arch wires. Clinical trial registration is found at RBR-7g5zng.

## 1. Introduction

Mandibular anterior crowding has been assigned to discrepancies in both tooth and mandibular size and the proportions of tooth size between maxillaries [1]. Tooth morphogenesis, the development of dentition, and the growth of the craniofacial complex are some of the factors that may be implicated in the origin of crowding, which has been studied in the attempt to define objectives and methods for efficient treatments.

Nickel-titanium (NiTi) alloys are widely used for alignment and leveling because of their low stiffness, which generates low intensity forces. NiTi heat-activated wires have two specific characteristics, shape memory and superelasticity, which result from the capacity of molecular changes when being under the effect of temperature and deflection [2, 3]. Superelasticity, combined with shape memory, which is inherent to these alloys, simplifies and shortens clinical treatments, as it decreases working time because it generates low intensity forces even when wire deflections are large [4].

In vitro and in vivo studies have been showing that NiTi wires optimize the tooth movement [5], since this material was included in the wire sequence for orthodontic treatment.

In vitro studies have clearly demonstrated that there are differences in the mechanical properties of conventional and NiTi heat-activated arch wires. Superelasticity in the hysteresis curve of NiTi heat-activated arch wires has a deactivation plateau that is typical of martensitic phase transformation. This plateau promotes constant forces, reported to be favorable to tooth movement, as long as they are low intensity forces [6–8]. However, few clinical studies have analyzed the efficacy and the mechanical properties of NiTi heat-activated wires, and the advantages of their clinical use remain controversial and inconclusive [9–11].

This study evaluated the time to correct mandibular crowding using two sequences, one of conventional NiTi arch wires and the other of conventional and NiTi heat-activated arch wires testing the null hypothesis that there was no significantly statistical difference between the two techniques.

## 2. Material and Methods

### 2.1. Sample Calculation

The sample calculation was carried out broaching the following characteristics: A two-sided log rank test with an overall sample size of 20 subjects (10 in the control group and 10 in the treatment group) achieves 0.50 power at a 0.05 significance level to detect a hazard ratio of 0.40 when the proportion surviving in the control group is 0.50. The study lasts for 6 time periods of which subject accrual (entry) occurs in the first time period (PASS 11, Kaysville, Utah, USA). Given the possibility of “dropouts” in the region of 10%, a total of 22 patients were selected.

This prospective randomized study was conducted from March 2011 to March 2012. Twenty-two boys and girls (mean age: 16.68 ± 2.66) were included in the study.

### 2.2. Patient Selection

Patients who accepted to participate in this study signed an informed consent term. The study was approved by the Ethics in Research Committee of Centro Universitário do Maranhão (UniCeuma) under number 00143/10 and registered at http://www.ensaiosclinicos.gov.br/ (RBR-7g5zng).

Clinical examinations and patient history were used to select patients, and gingivitis, caries, and other diseases were treated before appliance placement.

### 2.3. Inclusion Criteria

Inclusion criteria were moderate mandibular anterior crowding (3–6 mm) at baseline, according to the irregularity index described by Little [12]; all teeth being present in the mandibular arch, up to second molars; no prescription for extractions in the mandibular arch; no need for treatment with intermaxillary elastics, interproximal stripping, open NiTi compression springs, or active labial bow; no diseases that might affect tooth movement; and no significant tooth size and shape abnormalities.

Patients with a history of trauma and resorption that, for any reason, did not visit the clinic for more than a month, whose mandibular appliance broke, or who had undergone previous orthodontic treatment were excluded from the sample.

### 2.4. Randomization

Patients that met inclusion criteria and signed the informed consent term were randomized to two groups. Paper cards describing the technique to be applied were used for randomization: sequence 1: conventional NiTi arch wires; sequence 2: conventional and NiTi heat-activated arch wires, as recommended by Gurgel (2006) [13]. The cards were sealed in envelopes that were opened later, at the time of randomization. After randomization, the treatment followed a previously defined schedule, shown in Table 1.

The sequences proposed are based on previous studies [5, 9, 10], whose cross section was defined according to type of arch wire used (NiTi or NiTi heat-activated). Monthly exchanges of the arch wires were performed, because the moderate crowding [12] is appropriate and allowed to this clinical procedure.

### 2.5. Study Models

Before placement and after each arch wire change, impressions using Jeltrate Plus (Dentsply, Petrópolis, Brazil) were taken, and a plaster study model using Special Micrograin Type IV Dental Stone (Vigodent, Bonsucesso, Brazil) was obtained. Alignment and leveling were monitored for five months. The casts were numbered, and data were recorded in a table with date, sequence used, arch wire used, crowding index, and patient name and age. Crowding correction was evaluated only for mandibular anterior teeth, regardless of possible posterior crowding. At the end of the experimental period, patients and sequences used were identified for those cases in which crowding had not been corrected. Twenty-two patients were evaluated, 11 in each group, and 132 casts were obtained and measured.

### 2.6. Fixed Appliance

All patients were treated using a Roth fixed appliance, 022′′ slot (3M, São José do Rio Petro, Brazil) using conventional and NiTi heat-activated and stainless steel arch wires (Orthometric, Marília, Brazil) fixed with grey color ligature elastic (Morelli, Sorocaba, Brazil).

### 2.7. Crowding Index

The crowding index was defined according to measurements of the plaster model, according to the irregularity index defined by Little [12]. A digital caliper measuring to the nearest 0.01 mm (Mitutoyo Digimatic, Kyoto, Japan) was held parallel to the occlusal plane for measurements. The linear movement of anatomic contacts was measured between the following points: mesial aspect of left canine and distal aspect of left lateral incisor; mesial aspect of left lateral incisor and distal aspect of left central incisor; mesial aspect of left central incisor and mesial aspect of right central incisor; distal aspect of right central incisor and mesial aspect of right lateral incisor; distal aspect of right lateral incisor and mesial aspect of right canine. The sum of these five measurements was the irregularity index, as defined by Little.

### 2.8. Operator Calibration

A single examiner, not directly involved in patient treatment and blind to the type of treatment to which the patient was subjected, was trained and calibrated to make crowding measurements in the cast models. Calibration consisted of crowding measurements using five mandibular arch models at three time points at an interval of one week between them, when five measurements were made for each model, at a total of 75 measurements. Intraclass Correction (ICC) was used to calculate intraexaminer agreement, which was significant; that is, the measures followed the same pattern (ICC = 0.971; *p* < 0.001; 95% CI, 0.945–0.986).

### 2.9. Statistical Analysis

Crowding indices for each month were recorded using a Microsoft Excel 2007 for Windows spreadsheet (Microsoft Corporation, Redmond, USA). After data were recorded, sex, age, and mean and standard deviation values of crowding indices were described for each period under analysis.

A Kaplan-Meier survival curve was built to illustrate treatments completed for a long time, and mean time to treatment completion was estimated for each group. Treatments not completed during the study time were censored. Completed cases, that is, those that reached a crowding index equal to zero in five months, were included in the analysis. Time to treatment completion between groups was compared using the log rank test.

SPSS 21.0 (IBM, Armonk, NY, USA) was used for all statistical analyses. The level of significance was set at 5%.

## 3. Results

After a thorough examination, 22 patients were included at the beginning of the study. The mean age was 16.43 (±2.42) for the group with the NiTi sequence and 16.90 (±2.14) for the group with the NiTi heat-activated sequence. Mean values and standard deviations were similar for both genders.

Mean and standard deviation values of crowding index during treatment are shown in Table 2. Crowding indices decreased uniformly. Standard deviation values were similar for the conventional and NiTi heat-activated sequences at each time point.

Table 3 shows that the treatment of mandibular anterior crowding was complete in all cases in the group that used the NiTi heat-activated sequence at the end of the study time (5 months), whereas 30% of those that used the NiTi sequence were not complete.

**Figure 1:**
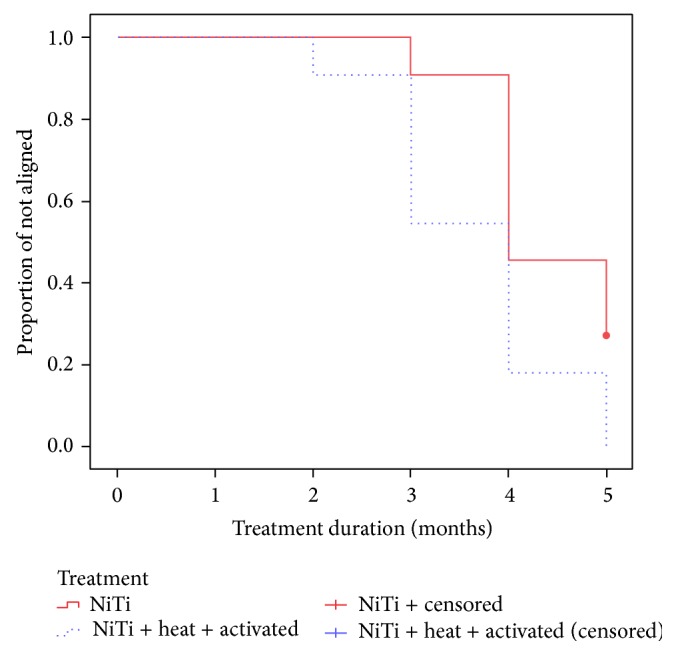
Kaplan-Meier survival estimates. Proportion of treatments not completed according to arch wire sequence.

There was a significant difference in time to achieve resolution between groups (log rank = 5.996, *p* < 0.05). The sequence that used heat-activated arch wires led to crowding resolution at a shorter time than the sequence of NiTi arch wires (Figure 1).

## 4. Discussion

In the present study, treatment for anteroinferior crowding was conducted using two clinical sequences incorporating NiTi and NiTi heat-activated wire. Patients with different degrees of crowding could complicate the baseline standardization; however only patients with a degree of crowding between 3 and 6 millimeters were recruited and, in addition, the patients' ages were similar, the respective means being 16.43 for Group I and 16.9 for Group II.

Moreover, as far as the methodology used in the present study is concerned, the patients were randomly divided into two groups and treated in accordance with a standard protocol. Care was taken to standardize the highest possible number of independent variables, just varying the wire sequence. One highly experienced operator was responsible for all clinical procedures. The examiner responsible for measuring the degree of crowding in each phase of treatment was blind as to the sequence used in the dental crowding treatment.

The variable of interest in this study was length of time from beginning of treatment to full correction of mandibular anterior crowding. Therefore, the level of crowding of patients in both groups had to be standardized, and only individuals with moderate crowding (3 to 6 mm) were included in the study. The group treated with conventional NiTi sequences had a mean crowding index of 4.37 mm (±0.80), whereas in the group that included NiTi heat-activated arch wires this index was 4.59 mm (±0.75). There was no significant difference between the groups (*p* = 0.51).

The null hypothesis was rejected, which demonstrated that the treatment with NiTi heat-activated arch wires achieved correction significantly more rapidly than the sequence using only conventional NiTi arch wires.

Mandibular crowding may be estimated using the irregularity index recommended by Little [12]. Previous studies found that this index is a reliable measure when compared with visual inspection or computer-assisted analysis [14] and that it may be used to standardize studies that investigate the initial, final, and postretention phases of the treatment of crowding. One of the limitations of this index is the fact that it is a measure of irregularity and, therefore, is not sensitive to tooth rotation and axial inclination [1, 15]. Moreover, it is not a measure of arch length but a guide to quantify mandibular anterior crowding. It was chosen for this study because it is simple, clinically safe, and reliable to evaluate dental crowding and because it has already been used in other studies [10, 11, 16].

In the method to asses efficacy used in this study (survival analysis), participants are classified as dropouts when they leave the study for any reason. In this study, no participant was classified as a dropout. In addition, survival analysis uses information about all participants up to the moment when they achieve the planned event or are censored, which is an ideal technique to analyze binary variables, such as crowding correction in our study. It is appropriate for longitudinal studies characterized by different lengths of follow-up for each participant and loss to follow-up. For these reasons, survival analysis was chosen to describe our results, and the log rank test was used to assess significance of the comparison between the two groups.

Our results demonstrated that the sequence that included NiTi heat-activated arch wires significantly improved the correction of mandibular anterior crowding when compared with the one that used only conventional NiTi arch wires. This result may be explained by the mechanical properties of superelasticity at oral temperatures and shape memory of the NiTi heat-activated wires. When heat-activated arch wires are used, movement forces of alignment and leveling are present during all the time between activations of the fixed appliance. Tooth movement is, therefore, more efficient because of the superelasticity plateau of the martensitic phase transformations [6].

Although laboratory studies have demonstrated the efficacy of materials characterized by superelasticity and shape memory [2], there is little clinical evidence in the literature supporting the advantages associated with these mechanical properties. Also, in clinical studies it was possible to observe that NiTi wire is more efficient for alignment procedure [5]. This in vivo study of crowding correction demonstrated this efficacy. The inclusion of heat-activated arch wires in a sequence of conventional orthodontic arch wires improved mandibular anterior crowding correction in our five-month study.

Continuous scientific development has brought innovation to NiTi wires, which can now be activated at temperatures close to those found in the oral cavity. NiTi wires that feature this specificity, called heat-activation, are highly flexible and superelastic [6]. With the advent of new alloys, stiffness of an orthodontic appliance can now be changed while the size and the cross-sectional dimensions of the wires remain the same. Therefore, a wide combination of different force magnitudes may be produced using wires of the same cross section but having a different modulus of elasticity.

Further studies should evaluate the effect that the variables under study here have on the clinical efficiency of planned sequences, as well as on other parameters, such as patient discomfort and incidence of resorption. They should also evaluate different NiTi wire brands, because, according to some authors [7, 8], the mechanical properties of NiTi wires vary significantly between manufacturers.

## 5. Conclusions

The sequence that included NiTi heat-activated wires achieved correction of mandibular anterior crowding more rapidly than the sequence that used only conventional NiTi wires. Five months after baseline assessment, mandibular anterior crowding was corrected in 100% of the cases whose treatment included NiTi heat-activated arch wires.

## Figures and Tables

**Table 1:** Arch wires (inches) used in each clinical sequence.

Schedule	Conventional NiTi arch wires	NiTi heat-activated arch wires
First month	.012′′ NiTi	
Second month	.016′′ NiTi	.018′′ NiTi heat-activated
Third month	.018′′ NiTi	.016′′ × .022′′ NiTi heat-activated
Fourth month	.019′′ × .025′′ NiTi	
Fifth month	.019′′ × .025′′ steel	

**Table 2:** Mean and standard deviation values of Little's index of irregularity during treatment phases.

Group	Baseline	1st assessment	2nd assessment	3rd assessment	4th assessment	5th assessment
NiTi sequence	4.37 (±0.80)	2.85 (±0.86)	1.82 (±0.83)	0.98 (±0.65)	0.38 (±0.64)	0.23 (±0.54)

NiTi heat-activated sequence	4.59 (±0.75)	2.63 (±0.66)	1.35 (±0.70)	0.48 (±0.55)	0.16 (±0.36)	0 (±0.00)

**Table 3:** Number (%) of cases completed.

Group	Assessment			
	2	3	4	5
NiTi sequence	—	1 (9.1%)	6 (54.5%)	8 (72.7%)

NiTi heat-activated sequence	1 (9.1%)	5 (45.5%)	9 (81.8%)	11 (100%)
